# Nodule on Apex of Root

**Published:** 1897-04

**Authors:** H. L Ambler

**Affiliations:** Cleveland, Ohio


### Nodule on Apex of Root.

Read before the American Dental Association.

BY H. L. AMBLER, D.D.S., M. D., CLEVELAND, OHIO.

A lady, aged about 30, presented for treatment of a chronic abscess of right superior lateral incisor, upon examination with probe it was found that the labial wall of bone, at the apex of the root, had been destroyed. With the intention of scraping the apex and breaking up the sac, a small right-angled scoop was introduced and passed easily over and around the apex. Upon withdrawing it, a small nodule of dentine or enamel, about the size of an ordinary pin-head, was brought away. The abscess was treated with pyrozone four times, at intervals of three days, when the discharge ceased. Evidently the pulp had been dead for a long time, and the canal filled, as there were large mesial and distal fillings. The case was lost sight of for a year, and in the meantime she had this lateral, and also the right central, which was badly carious, extracted, and is now wearing an artificial denture. The special interest attached to the case is the fact of finding a nodule at the apex of the root, especially of a single-rooted tooth.

Enamel-nodules are small excrescences, apparently consisting of enamel, occasionally met with upon the roots of teeth. They are generally found upon the multi-rooted teeth, situated a little below the neck and often at the junction of two roots. On section they are found to consist of a cone of dentine covered with a rather thick layer of enamel, and often connected to the crown with enamel.

Wedl says that these nodules are the result of localized continuations of development of the enamel between the already-developed basal portion of the roots, and are produced by the strip of the enamel organ which has persisted longer than the rest. Smale shows a cut of one of these nodules on the apex of a molar root, and says they may be accounted for by a budding from the tissues concerned in the process of the formation of the tooth. Tomes shows a cut of a nodule situated on the neck of a single-rooted superior cuspid.

### Report of Committee on Nomenclature.

Read before the American Dental Association, August 4th, 1896.

